# Genetic susceptibility to multiple sclerosis in African Americans

**DOI:** 10.1371/journal.pone.0254945

**Published:** 2021-08-09

**Authors:** Douglas S. Goodin, Jorge R. Oksenberg, Venceslas Douillard, Pierre-Antoine Gourraud, Nicolas Vince

**Affiliations:** 1 Department of Neurology, University of California, San Francisco, CA, United States of America; 2 Université de Nantes, CHU Nantes, Inserm, Centre de Recherche en Transplantation et Immunologie, UMR 1064, ITUN, Nantes, France; Oklahoma Medical Research Foundation, UNITED STATES

## Abstract

**Objective:**

To explore the nature of genetic-susceptibility to multiple sclerosis (MS) in African-Americans.

**Background:**

Recently, the number of genetic-associations with MS has exploded although the MS-associations of specific haplotypes within the major histocompatibility complex (*MHC*) have been known for decades. For example, the haplotypes HLA-DRB1*15:01~HLA-DQB1*06:*02*, and HLA-DRB1*03:01~ HLA-DQB1*02:*01* have odds ratios (*ORs*) for an MS-association orders of magnitude stronger than many of these newly-discovered associations. Nevertheless, all these haplotypes are part of much larger conserved extended haplotypes (*CEHs*), which span both the Class I and Class II *MH*C regions. African-Americans are at greater risk of developing MS compared to a native Africans but at lesser risk compared to Europeans. It is the purpose of this manuscript to explore the relationship between MS-susceptibility and the *CEH* make-up of our African-American cohort.

**Design/methods:**

The African-American (AA) cohort consisted of 1,305 patients with MS and 1,155 controls, who self-identified as being African-American. For comparison, we used the 18,492 controls and 11,144 MS-cases from the predominantly European Wellcome Trust Case Control Consortium (WTCCC) and the 28,557 phased native Africans from the multinational “Be the Match” registry. The WTCCC and the African-Americans were phased at each of five *HLA* loci (*HLA-A*, *HLA-C*, *HLA-B*, *HLA-DRB1* and *HLA-DQB1*) and the at 11 *SNP*s (10 of which were in non-coding regions) surrounding the Class II region of the *DRB1* gene using previously-published probabilistic phasing algorithms.

**Results:**

Of the 32 most frequent *CEHs*, 18 (56%) occurred either more frequently or exclusively in Africans) whereas 9 (28%) occurred more frequently or exclusively in Europeans. The remaining 5 *CEHs* occurred in neither control group although, likely, these were African in origin. Eight of these *CEHs* carried the DRB1*15:03~DQB1*06:*02~a36* haplotype and three carried the DRB1*15:01~DQB1*06:*02~a1* haplotype. In African Americans, a single-copy of the European *CEH* (*03*:*01_07*:*02_07*:*02_15*:*01_06*:*02_a1*) was associated with considerable MS-risk (*OR = 3*.*30; p = 0*.*0001*)–similar to that observed in the WTCCC (*OR = 3*.*25; p<10*^−*168*^*)*. By contrast, the MS-risk for the European *CEH* (*02*:*01_07*:*02_07*:*02_15*:*01_06*:*02_a1*) was less (*OR =* 1.49; *ns*)–again, similar to the WTCCC (*OR = 2*.*2; p<10*^−*38*^). Moreover, four African haplotypes were “protective” relative to a neutral reference, to three European *CEHs*, and also to the five other African *CEHs*.

**Conclusions:**

The common *CEHs* in African Americans are divisible into those that are either African or European in origin, which are derived without modification from their source population. European *CEHs*, linked to MS-risk, in general, had similar impacts in African-Americans as they did in Europeans. By contrast, African *CEHs* had mixed MS-risks. For a few, the MS-risk exceeded that in a neutral-reference group whereas, for many others, these *CEHs* were “protective”–perhaps providing a partial rationale for the lower MS-risk in African-Americans compared to European-Americans.

## Introduction

The pathogenesis of multiple sclerosis (MS) is complex and the susceptibility of an individual to developing this disease depends critically upon both environmental events and genetic factors [1–5]. Recently, a great deal of progress has been made with regard to our understanding of both aspects of MS pathogenesis. On the genetic side, for example, 233 loci, in diverse genomic regions, have now been identified as MS-associated by genome-wide association screens (*GWAS*), which use large arrays of single nucleotide polymorphisms (*SNPs*) scattered throughout the genome [4, 6–14]. Many of these implicated regions are located within or close to immune-related genes, which are involved in either the adaptive or innate arms of the human immune system [4]. Moreover, 32 of these independently MS-associated *SNPs* are located within the major histocompatibility complex (*MHC*) on the short arm of Chromosome 6 [4]. Despite this recent increase in the number and location of these MS-associations, however, certain human leukocyte antigens (*HLA*), located within the *MHC*, have long been known to be MS-associated [12, 15–22]. For example, among persons of European descent, the Class II HLA-DRB1*15 alleles have been known for decades to have a strong MS-association. In addition, the relationship of other *HLA* alleles (either to MS or to other diseases) has also been well-established [12, 15–23]. Typically, these studies have been focused on identifying the relationship of genetic susceptibility to specific alleles at specific *HLA* loci. For example, in individuals of European descent, the focus has been on the increased “risk” associated with carrying either HLA-DRB1*15:*01* or HLA-DRB1*03:*01* alleles and on the “protective” effect of carrying the HLA-A*02:*01* allele [15–25]. For example, in the Wellcome Trust Case Control Consortium (WTCCC) dataset [13], the odds ratio (*OR*) of MS for individuals possessing one or more of these alleles is highly significant–for HLA-DRB1*15:*01* (*OR = 3*.*24; p<<10*^−*300*^); for HLA-DRB1*03:*01* (*OR = 1*.*27; p<10*^−*11*^); and for HLA-A*02:*01* (*OR = 0*.*69; p<10*^−*53*^).

Despite this focus on single alleles of specific genes, however, these *HLA* alleles don’t really exist in isolation. Indeed, it has been known for decades that multiple *HLA* alleles within both the Class I and II regions of the *MHC* influence, often interactively, the risk of developing MS [26]. For example, within the *MHC*, most *HLA* alleles are in tight linkage disequilibrium with each other and, overall, the *HLA* region consists of a relatively small collection of highly conserved extended haplotypes (*CEHs*), which stretch (at least) across the “classical” *HLA* genes (*HLA-A*, *HLA-C*, *HLA-B*, *HLA-DRB1*, and *HLA-DQB1*)–a distance spanning nearly 3 *mb* of DNA [27–31]. This haplotypic structure is found in all human populations, including Africans and persons of European descent [28]. Nevertheless, the *CEH* compositions, which account for this population structure, vary markedly between different regions [26–29]. Thus, in the predominantly European WTCCC, the most frequent 250 *CEHs* accounted for 57% of all *CEHs* present [29] and, in an African population [28], the most frequent 250 *CEHs* accounted for 31% of all *CEHs* present reflecting greater haplotypic diversity. Nevertheless, only 19 (4.0%) of these 500 “most-frequent” *CEHs* were shared as “most frequent” between the two populations. Thus, it seems that these *CEHs* are under a strong selection pressure, presumably based upon favorable biological properties of the complete haplotypes in certain environments [27–31].

In the *HLA* Class II region, this linkage disequilibrium is especially strong between (at least) between the *HLA-DRB1* and *HLA-DQB1* loci. For example, in the predominantly European data from the WTCCC, 97.5% of the HLA-DRB1*15:*01* alleles (the most common *DRB1* allele in Europeans; control frequency = 13.0%) are linked to the HLA-DQB1*06:*02* allele. Similarly, 98.4% of the HLA-DRB1*03:*01* alleles (control frequency = 11.8%) are linked to the HLA-DQB1*02:*01* allele. The same is true in an African population [27]. Thus, in Africans, 98.3% of the HLA-DRB1*15:*01* alleles (control frequency = 1.8%) are similarly linked to HLA-DQB1*06:*02* allele and 99.5% of HLA-DRB1*03:*01* alleles (control frequency = 7.3%) are also linked to the HLA-DQB1*02:*01* allele [27]. Moreover, in Africans, 98.9% of the HLA-DRB1*15:*03* alleles (the most common *DRB1* allele in Africans; control frequency = 12.5%) are linked to the HLA-DQB1*06:*02* allele. Similar tight linkages are found for most other *DRB1~DQB1* combinations [29]. In addition, we have described a collection of *SNP*-haplotypes that are composed of unique combinations of the 11 *SNPs* (*rs2395173*; *rs2395174*; *rs3129871*; *rs7192*; *rs3129890*; *rs9268832*; *rs532098*; *rs17533090*; *rs2187668*; *rs1063355*; and *rs9275141*), and which span 0.25 *mb* of DNA surrounding the *HLA-DRB1* locus [29]. Ten of these *SNPs* are within intergenic regions whereas *rs1063355* is within exon 5 of the *DQB1* gene. One such 11-*SNP* haplotype (*a1*), adds further specificity to the HLA-DRB1*15:01~HLA-DQB1*06:*02* haplotype [29]. Thus, 99% of (*a1*) *SNP*-haplotypes carry the HLA-DRB1*15:01~HLA-DQB1*06:*02* haplotype and, conversely, 99% of these *HLA-*haplotypes carry the (*a1*) *SNP*-haplotype [29]. This complete *HLA* Class II haplotype (DRB1*15:01~DQB1*06:*02~a1*) is referred to as the (*H+*) haplotype.

Regardless of such strong linkage disequilibrium in the Class II region, however, there are nuances to susceptibility that accrues because of the *CEH* structure. For example, in persons of European descent, the Class II HLA-DRB1*03:01~ HLA-DQB1*02:*01* haplotype comes in two forms. The first (present in 84% of the WTCCC controls) is coupled to the (*a6*) *SNP*-haplotype and the second (present in 15% of the WTCCC controls) is coupled to the (*a2*) *SNP*-haplotype [29]. Each form has a distinct relationship to susceptibility. For (*a2*) carriers, among non-(*H+*)-carrying individuals, a single copy is consistently associated with an increased MS-risk [29]. By contrast, for (*a6*) carriers, the risk associated with carrying a single copy varies from being associated with “risk” to being “protective” depending upon the Class I portion of the *CEH* being considered [29]. Similarly, all carriers of the (*H+*) haplotype have an increased MS-risk, although the degree of association varies depending upon the *CEH* involved [29]. By contrast, some HLA-DRB1*15:01~ HLA-DQB1*06:*02* haplotypes that don’t also carry the (*a1*) *SNP*-haplotype, seem not to be associated with any MS-risk [29]. And, finally, although the HLA-A*02:*01* allele is “protective” when considered as a single allele, some of the *CEHs* on which this allele is present seem to have little impact on MS-risk whereas on other *CEHs* this allele seems to have a “protective” effect [29].

Given this strong linkage disequilibrium it is unclear what gene (or genes) within a “risk” haplotype is responsible for the increased susceptibility to MS that is observed. We have previously reported that, in an African American population, both the HLA-DRB1*15:*01* and the HLA-DRB1*15:*03* alleles (in the absence of the HLA-DQB1*06:*02* allele) are associated with an increase in MS risk whereas the HLA-DQB1*06:*02* allele (in the absence of the HLA-DRB1*15 alleles) is not [32]. A similar observation is noted in the WTCCC data where HLA-DRB1*15:*01* in the absence of HLA-DQB1*06:*02* is associated with MS (*OR = 1*.*7; p = 0*.*0002*) whereas HLA-DQB1*06:*02* in the absence of HLA-DRB1*15:01 is not (*OR = 1*.*2; ns*). This asymmetry between loci the has been taken as evidence to suggest that MS susceptibility is related to something that lies telomeric to the *DQB1* locus, possibly at the *DRB1* locus itself [32]. Notably, however, the difference in *OR* between these two WTCCC observations is not significant (*p = 0*.*11*). In addition, another study utilized the fact that some African Americans lack the *HLA-DRB5* gene (telomeric to *DRB1*) and demonstrated that MS-susceptibility was unchanged in individuals who were missing this gene [33]. This observation was interpreted as supporting the notion that MS susceptibility could be mapped to the *DRB1* locus, although others have reported that *DRB5*, itself, may be related either to progression or susceptibility [34, 35]. Nevertheless, in this study [33], the authors also identified a single *SNP* (*rs1035798*), located in the region of the Class III *AGER* gene (telomeric to *DRB5*), which was independently associated with MS–i.e., when all carriers of DRB1*15 and DRB1*03 alleles were excluded from the analysis (*OR = 1*.*85; p = 0*.*008*). Similarly, the IMSGC reported 32 independent signals within the *MHC* [4].

It is unclear, however, given the haplotypic (*CEH*) structure of the *MHC*, whether these observations actually support any single gene (e.g., *DRB1*) as being responsible for the observed changes in MS-susceptibility. For example, using well-established MS epidemiologic parameters (e.g., the disease prevalence, the proportion of women among MS patients, the recurrence-risks for MS in siblings and twins of an MS proband, and the time-dependent changes in the sex-ratio) and based theoretical considerations, less than 7.3% of the general populations of North America and Europe have any chance, whatsoever, of getting MS [5]. Therefore, because 23% of controls in the WTCCC carry one or more copies of the DRB1*15:01~HLA-DBQ1*06:*02~a1* or (*H+*) haplotype, this indicates that fewer than 32% (7.3/23) of these (*H+*)-carrying individuals have any chance at all of getting the disease–i.e., more than 68% of (*H+*)-carrying individuals have no chance of developing MS, regardless of their environmental experiences [5]. Moreover, as noted above, some carriers of the HLA-DRB1*15:01~HLA-DBQ1*06:*02* haplotype, but not carriers of (*a1*), seem to have little, if any, MS-risk [29]. From these considerations, it seems clear that *CEH* composition within a population is a critical factor for MS pathogenies. It is the purpose of this manuscript, therefore, to explore the relationship between MS susceptibility and the *CEH* composition of our African American (AA) cohort.

## Results

**Fig 1:**
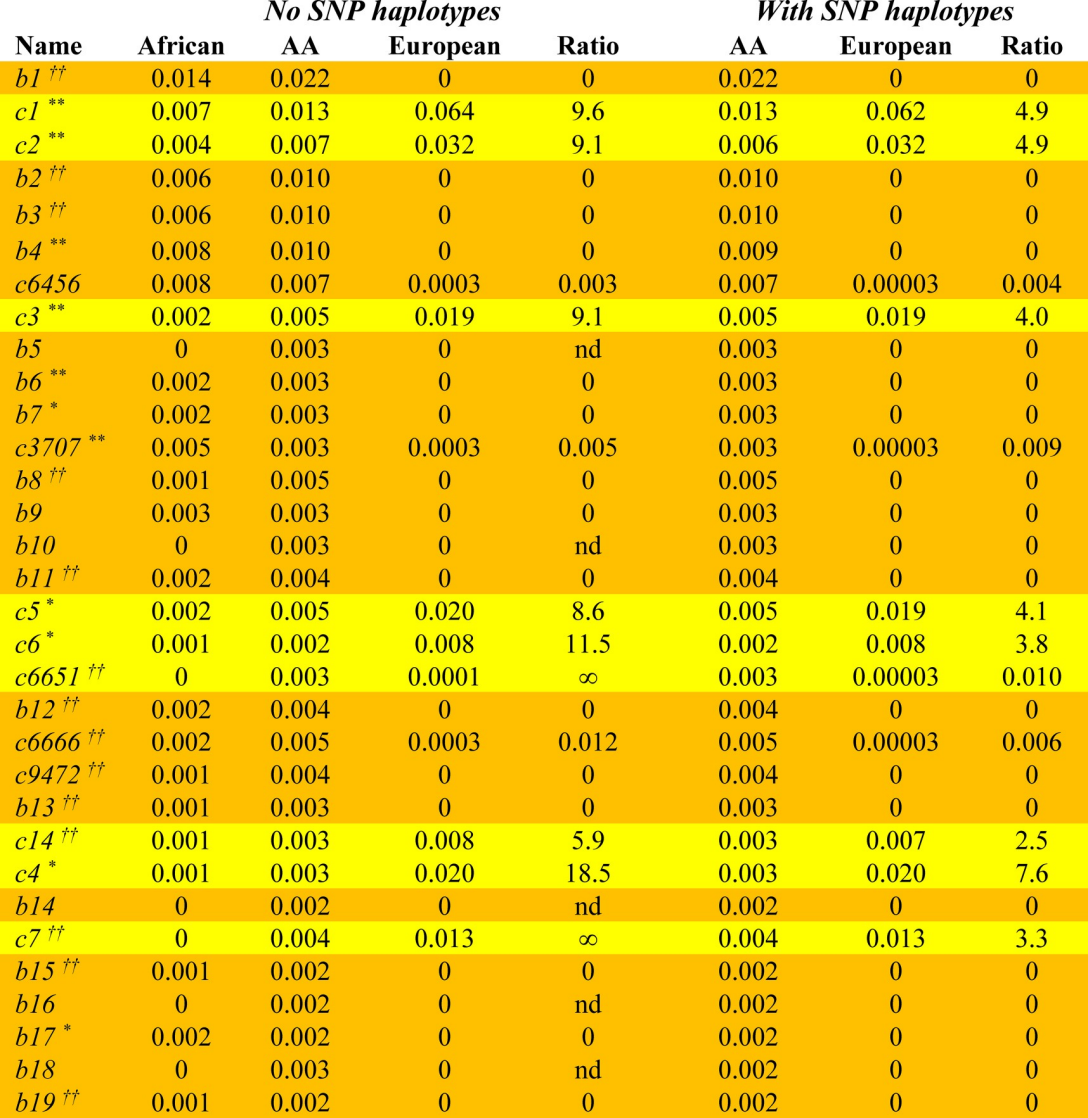
*CEH* frequency in African, European, and African American control populations. African control frequencies are from Gragert, et al. [26], European control frequencies are from the WTCCC [13, 14], and AA control frequencies are from the AA dataset reported here. African *CEHs* are highlighted in orange whereas European *CEHs* are highlighted in yellow (*see Text*). Only *CEHs* with ≥12 representations in the AA cohort are listed. The “*No SNP haplotypes*” condition is for *CEHs* not including any associated *SNP*-haplotype (*Tables 1 & 2*). The “*With SNP haplotypes*” condition is for *CEHs* that include the associated *SNP*-haplotype as indicated in *Tables 1 & 2*. Name indicates the haplotype (*Tables 1 & 2*), sorted in descending order of frequency in the WTCCC [13, 29]–designated by (**c**)–and in the AA cohort for *CEHs* not found in the WTCCC–designated by (**b**). In the *Table*, (0) indicates true zero. Ratios are of *CEH* frequency in Europeans to that in Africans (No *SNP*s condition) and the frequency ratio in Europeans to that in African Americans (With *SNPs* condition). nd = not defined; ∞ = infinity. Significance of the difference in *CEH* frequency between Africans and Europeans are indicated as follows:
*    *    10^−4^ ≤ p < 0.05***    10^−8^ ≤ p < 10^−4^†*    *    10^−12^ ≤ p < 10^−8^††*    p < 10^−12^
It appears that the Class II haplotypes DRB1*07:01_DQB1*02:*02* and DRB1*09:01_DQB1*02:*02* in the WTCCC data are consistently identified in both African and European populations as DRB1*07:01_DQB1*02:*01* and DRB1*09:01_DQB1*02:*01*, respectively, in the data of Gragert and colleagues [28]–*see Text*. If these Class II haplotypes are, in fact, the same, there is still a significantly greater (*c7*) *CEH* frequency in Europeans compared to Africans (*p*<10^−8^). However, in this case, the (*b14*) *CEH* occurs only in Africans, whereas the (*b10*) *CEH* still occurs in neither Africans nor Europeans.

**Fig 2:**
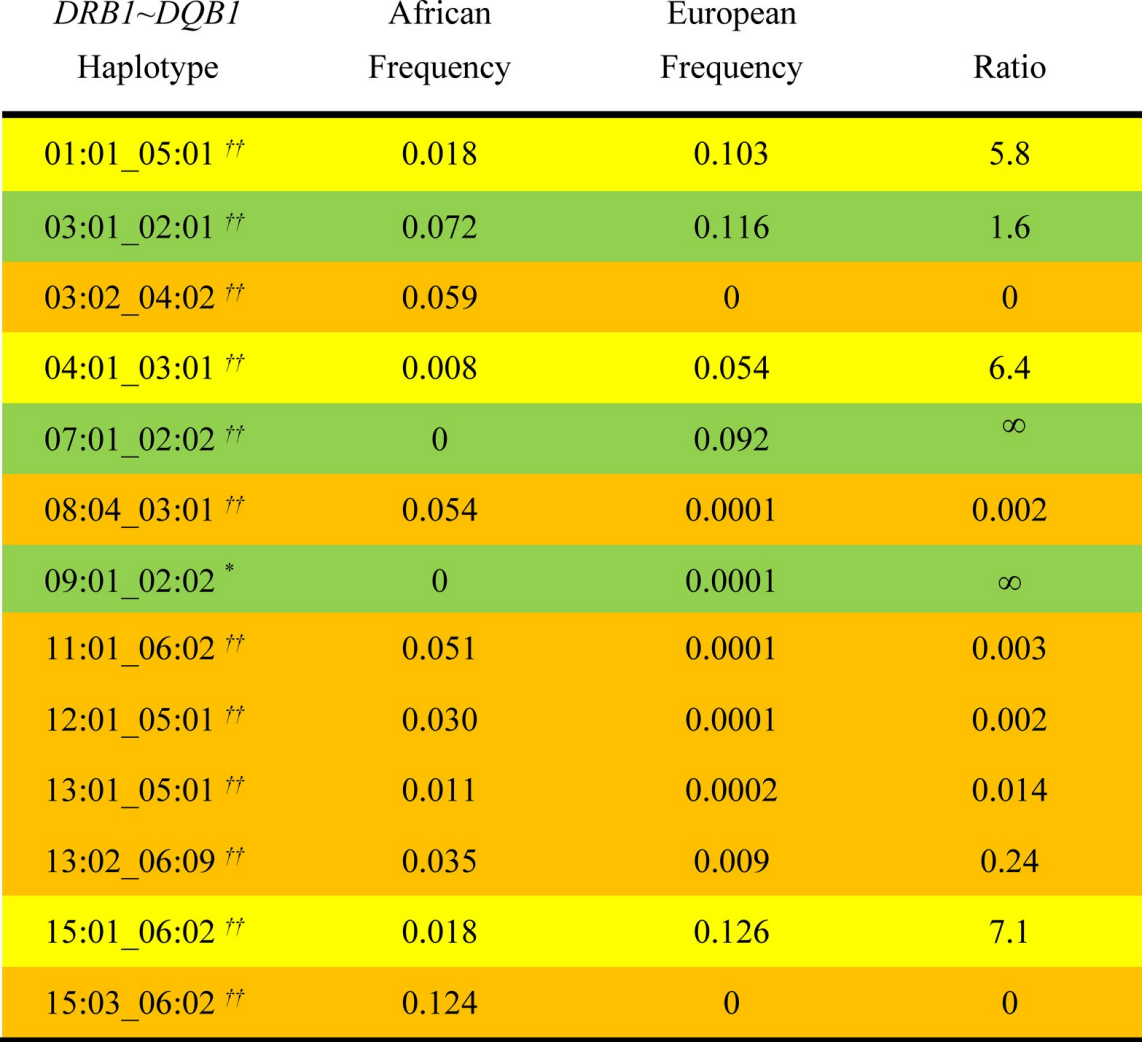
*DRB1~DQB1* Class II haplotype frequencies in Africans and Europeans control populations. African control frequencies are taken from Gragert, et al. [28] whereas European control frequencies are taken from the WTCCC [13, 29]. Predominantly African haplotypes are highlighted in orange whereas predominantly European haplotypes are highlighted in yellow (*see Text*). Haplotype of uncertain origin, either because of a similar frequency in both groups (*03*:*01_02*:*01*) or because of ambiguities (*see below*) are highlighted in green. In the *Table*, (0) indicates true zero. Only Class II haplotypes for the *CEHs* with ≥12 representations in the AA cohort (*see Fig 3*) are listed. Ratios are of the haplotype frequency in Europeans controls (from the WTCCC) to that in Africans. ∞ = infinity. Significance of the difference in Class II haplotype frequency between Africans and Europeans are indicated as follows:
*        10^−4^ ≤ p < 0.05**    10^−8^ ≤ p < 10^−4^†        10^−12^ ≤ p < 10^−8^††*    p < 10^−12^
As noted in the legend of *Fig 1*, it appears that the Class II haplotypes DRB1*07:01_DQB1*02:*02* and DRB1*09:01_DQB1*02:*02* in the WTCCC data are consistently identified in both African and European populations as DRB1*07:01_DQB1*02:*01* and DRB1*09:01_DQB1*02:*01*, respectively, in the data of Gragert and colleagues [28]–*see Text*. If these Class II haplotypes are, in fact, the same, there is no significant difference in the frequency of the Class II haplotype DRB1*07:01_DQB1*02:*01* in Africans (9.6%) and the frequency of DRB1*07:01_DQB1*02:*02* in Europeans (9.2%). By contrast, in this case, the Class II haplotype DRB1*09:01_DQB1*02:01* is significantly more common in Africans (2.7%) than the haplotype DRB1*07:01_DQB1*02:*02* in Europeans (0.01%)–*p* < 10^−12^.

Among the 2,460 African American individuals in this study, there were 4,920 total *CEHs* present, of which 2,744 were unique and, of these, 679 had more than one representation in the dataset. The 32 *CEHs* having at least 12 representations accounted for 16% of the total number of *CEHs* present (*Tables 1 and 2*) and, moreover, the 250 most frequently occurring *CEHs* accounted for 39% of the total. In addition, the likely source of these *CEHs* in the admixture (African or European) seemed, for the most part, clear because they remained unaltered in the AA cohort when compared either to their exclusive source population or to both populations. For example, of these 32 most frequent *CEHs*, 18 (56%) occurred either much more frequently or exclusively in an African compared to a European population (*Fig 1*) whereas 9 (28%) occurred much more frequently or exclusively in a European population. In all of these cases, the full haplotype was represented in the reference populations (*Fig 1*). The remaining 5 *CEHs* of *Fig 1* were not found in either the African or the European control populations. Because (*b5*), (*b16*), and (*b18*) carry the predominantly (or exclusively) African *HLA* Class II motifs of DRB1*15:03~DQB1*06:*02* or DRB1*13:01~DQB1*05:*01*, these *CEHs* seem likely to be of African origin (*Fig 2*). Because (*b10*) and (*b14*) carry the (apparently exclusive) European *HLA* Class II motifs of DRB1*07:01~DQB1*02:*02* or DRB1*09:01~DQB1*02:*02*, these *CEHs* might seem likely to be of European origin (*Fig 2*).

**Table 1:** *CEHs* in the AA population, which include either the *(H+)* haplotype or the DRB1*15:03~DQB1*06:*02* Class II haplotype ^†^.

	*HLA Haplotype*			
Name^††^	*A~C~B~DRB1~DQB1~SNP*	*OR1 (CI)* *	Percentage*	p–value**
*c2*	*03*:*01_07*:*02_07*:*02_15*:*01_06*:*02_a1*	3.3 (1.7–6.7)	0.6%	0.00005
*b3*	*34*:*02_04*:*01_44*:*03_15*:*03_06*:*02_a36*	0.5 (0.2–1.1)	1.0%	0.06
*c3*	*02*:*01_07*:*02_07*:*02_15*:*01_06*:*02_a1*	1.5 (0.6–3.7)	0.5%	0.33
*b5*	*66*:*02_07*:*18_58*:*01_15*:*03_06*:*02_a36*	2.0 (0.8–5.9)	0.3%	0.14
*b6*	*30*:*02_08*:*02_14*:*02_15*:*03_06*:*02_a36*	1.9 (0.7–5.5)	0.3%	0.19
*b7*	*68*:*02_04*:*01_53*:*01_15*:*03_06*:*02_a36*	1.7 (0.6–5.2)	0.3%	0.27
*b9*	*68*:*02_07*:*02_07*:*02_15*:*03_06*:*02_a36*	2.0 (0.7–6.6)	0.3%	0.17
*c6*	*24*:*02_07*:*02_07*:*02_15*:*01_06*:*02_a1*	2.2 (0.7–8.1)	0.4%	0.15
*b11*	*36*:*01_04*:*01_53*:*01_15*:*03_06*:*02_a36*	0.7 (0.2–1.9)	0.2%	0.47
*b12*	*30*:*01_17*:*01_42*:*01_15*:*03_06*:*02_a36*	0.7 (0.2–2.2)	0.4%	0.62
*b15*	*01*:*02_07*:*01_49*:*01_15*:*03_06*:*02_a59*	1.3 (0.4–5.2)	0.2%	0.78
*b18*	*74*:*01_02*:*10_15*:*03_15*:*03_06*:*02_a36*	1.3 (0.4–5.2)	0.2%	0.78
*b19*	*74*:*01_04*:*01_53*:*01_15*:*03_06*:*02_a36*	1.3 (0.4–5.2)	0.2%	0.78

† *CEHs* either carrying the DRB1*15:01 ~DQB1*06:*02~a1* haplotype–i.e., the (*H+*) haplotype)–or carrying the DRB1*15:03 ~DQB1*06:*02* haplotype. The listed *CEHs* are all of those that have ≥12 representations in the AA cohort.

†† Arbitrary name for haplotype, sorted in descending order of frequency in the WTCCC [3, 29]–designated by (**c**)–and in the AA cohort for *CEHs* not found in the WTCCC–designated by (**b**).

* Odds ratio (*OR1*) of disease for individuals having 1 copy of the listed *CEH* compared to a neutral reference group consisting of individuals having either no copies or no other copies of the (*H+*) haplotype (see Methods). The 95% confidence interval (*CI*) is in parenthesis. Percentage indicates the % of all *CEHs* in the AA Control population.

** The p-values for the *OR1* comparing cases to controls.

**Table 2:** Other common *CEH*s in the AA population^†^.

	*HLA Haplotype*			
Name^††^	*A~C~B~DRB1~DQB1~SNP*	*OR1 (CI)* *	Percentage*	p–value**
*b1*	*30*:*01_17*:*01_42*:*01_03*:*02_04*:*02_a93*	0.6 (0.4–1.0)	2.2%	0.06
*c1*	*01*:*01_07*:*01_08*:*01_03*:*01_02*:*01_a6*	1.5 (0.9–2.5)	1.3%	0.12
*b2*	*68*:*01_06*:*02_58*:*02_12*:*01_05*:*01_a61*	0.9 (0.4–1.8)	1.0%	0.75
*b4*	*33*:*03_04*:*01_53*:*01_08*:*04_03*:*01_a16*	0.5 (0.2–1.1)	0.9%	0.05
*c6456*	*36*:*01_04*:*01_53*:*01_11*:*01_06*:*02_a79*	0.5 (0.2–1.3)	0.7%	0.17
*c3707*	*68*:*02_03*:*04_15*:*10_03*:*01_02*:*01_a2*	1.9 (0.7–5.5)	0.3%	0.19
*b8*	*30*:*01_17*:*01_42*:*02_08*:*04_03*:*01_a49*	0.8 (0.3–2.0)	0.5%	0.65
*c5*	*02*:*01_05*:*01_44*:*02_04*:*01_03*:*01_a3*	0.6 (0.2–1.7)	0.3%	0.32
*b10*	*23*:*01_02*:*10_15*:*03_07*:*01_02*:*02_a3*	1.2 (0.4–3.4)	0.5%	0.82
*c6651*	*74*:*01_02*:*10_15*:*03_13*:*02_06*:*09_a25*	1.4 (0.4–4.8)	0.3%	0.61
*c6666*	*33*:*03_14*:*02_15*:*16_01*:*02_05*:*01_a23*	0.3 (0.1–1.1)	0.5%	0.07
*c9472*	*68*:*02_03*:*04_15*:*10_01*:*02_05*:*01_a23*	0.6 (0.2–2.0)	0.4%	0.44
*c14*	*02*:*01_07*:*01_08*:*01_03*:*01_02*:*01_a6*	0.9 (0.2–3.5)	0.3%	1.00
*c4*	*03*:*01_04*:*01_35*:*01_01*:*01_05*:*01_a9*	0.9 (0.2–3.5)	0.3%	1.00
*b13*	*68*:*02_03*:*04_15*:*10_08*:*04_03*:*01_a16*	0.8 (0.2–2.8)	0.3%	0.78
*c7*	*29*:*02_16*:*01_44*:*03_07*:*01_02*:*02_a5*	0.5 (0.1–1.7)	0.2%	0.25
*b14*	*30*:*02_07*:*02_07*:*02_09*:*01_02*:*02_a3*	2.1 (0.6–9.4)	0.4%	0.27
*b16*	*30*:*02_18*:*02_57*:*03_13*:*01_05*:*01_a10*	1.3 (0.4–5.2)	0.2%	0.78
*b17*	*68*:*02_17*:*01_42*:*01_03*:*02_04*:*02_a93*	0.9 (0.2–4.1)	0.2%	1.00

† *CEHs* not carrying either the (*H+*) haplotype (*see Table 1*) or the DRB1*15:03 ~DQB1*06:*02* haplotype and also having ≥ 12 representations in the AA cohort.

†† Arbitrary name for haplotype, sorted in descending order of frequency in the WTCCC [13, 29]–designated by (**c**)–and in the AA cohort for *CEHs* not found in the WTCCC–designated by (**b**).

* Odds ratio (*OR1*) of disease for individuals having 1 copy of the listed *CEH* compared to a neutral reference group consisting of individuals having no copies of that particular *CEH* and also no copies of any (*H+*) carrying *CEH* (see Methods). The 95% confidence interval (*CI*) is in parenthesis. Percentage indicates the % of all *CEHs* in the AA control population.

** The p-values for the *OR1* comparing cases to controls.

Nevertheless, this is probably not the case. For example, it appears that the Class II haplotypes DRB1*07:01_DQB1*02:*02* and DRB1*09:01_DQB1*02:*02* in both the WTCCC and AA datasets are consistently identified as DRB1*07:01_DQB1*02:*01* and DRB1*09:01_DQB1*02:*01*, respectively, in the data of Gragert and colleagues for both Africans and Europeans [28]. Thus, among the 2.4 million European Class II haplotypes in the “*Be the Match*” registry, there were no DRB1*07:01_DQB1*02:*02* haplotypes whereas the DRB1*07:01_DQB1*02:*01* haplotype accounted for 9.6% of all haplotypes present. By contrast, in the European WTCCC data here were no DRB1*07:01_DQB1*02:*01* haplotypes whereas the DRB1*07:01_DQB1*02:*02* haplotype accounted for 9.2% of all haplotypes present (*Fig 2*). Moreover, among the 28,557 Africans in the data of Gragert and colleagues [28], there were no DRB1*07:01_DQB1*02:*02* haplotypes whereas the DRB1*07:01_DQB1*02:*01* haplotype accounted for 9.6% of all haplotypes present. By contrast, in the AA dataset, there were no DRB1*07:01_DQB1*02:*01* haplotypes whereas the DRB1*07:01_DQB1*02:*02* haplotype accounted for 9.6% of all haplotypes present.

Similarly, among the Class II haplotypes in the “*Be the Match*” registry, among Europeans, there were no DRB1*09:01_DQB1*02:*02* haplotypes whereas the DRB1*09:01_DQB1*02:*01* haplotype accounted for 0.04% of all haplotypes present. By contrast, in the WTCCC data here were no DRB1*09:01_DQB1*02:*01* haplotypes whereas the DRB1*09:01_DQB1*02:*02* haplotype accounted for 0.01% of all haplotypes present (*Fig 2*). Moreover, in the “*Be the Match*” registry, among Africans, there were no DRB1*09:01_DQB1*02:*02* haplotypes whereas the DRB1*09:01_DQB1*02:*01* haplotype accounted for 2.7% of all haplotypes present. By contrast, in the AA dataset, there were no DRB1*09:01_DQB1*02:*01* haplotypes whereas the DRB1*09:01_DQB1*02:*02* haplotype accounted for 2.0% of all haplotypes present.

Although the same haplotype confusion seems to apply to other rare *CEHs* (i.e., not listed in *Table 2*), which carry the DQB1*02:*02* allele in the AA dataset, these differences cannot simply be attributed to a general typing difference for the DQB1*02:*01* and DQB1*02:*02* alleles between these different sets of data. Thus, in each of these datasets, only the very common haplotype HLA-DRB1*03:01_DQB1*02:*01* haplotype was represented. No dataset had any examples of a HLA-DRB1*03:01_DQB1*02:*02* haplotype.

If, as suggested from above, these Class II haplotype pairs are, in fact, the same, then the (*c7*) *CEH* occurs in both groups although still more commonly in Europeans (*p* < 10^−8^), the (*b10*) *CEH* still occurs in neither population, and the (*b14*) *CEH* occurs in only Africans (*Fig 1*). In addition, the DRB1*07:01_DQB1*02:*01* haplotype is slightly, but not significantly, more common Africans (9.6%) than the DRB1*07:01_DQB1*02:*02* haplotype is in Europeans (9.2%), whereas the DRB1*09:01_DQB1*02:*01* haplotype is significantly (*p* < 10^−12^) more common in Africans compared to the DRB1*09:01_DQB1*02:*02* haplotype in Europeans (*Fig 2*).

Moreover, of the 364 AA individuals judged by admixture to be 99.999% African, 13 (3.6%) carried at least one of these 5 unknown *CEHs* and all of these full *CEHs* were carried by at least one of these “African” AA individuals. In addition, none of these 364 AA individuals carried any of the “European” *CEHs* listed in *Fig 1*. Conversely, of the 40 AA individuals judged by admixture to be 99.999% European, no one carried any of these 5 *CEHs* and, also, no one had any of the “African” *CEHs* listed in *Fig 1*. In addition, of the1.24 million European individuals in the “*Be the Match*” registry [28], even considering the possible haplotype confusion (*described above*), 4 of these 5 *CEHs* were not carried by anyone and (*b14*) was still significantly more common among Africans (*p < 10*^*−12*^).

Taken together, this evidence suggests that each of these 5 *CEHs* are of African in origin and that, like the other frequent *CEHs* that we observed in this study, these *CEHs* have remained intact (unaltered) during the period of admixture. This breakdowns for *CEH* origin is also fully consistent with the average admixture (~73% African) that we observed in this cohort.

As noted above, the DRB1*15:03~DQB1*06:*02* haplotype is the most common haplotype among Africans. In our AA cohort, 87% of these *HLA* Class II haplotypes are linked to the (*a36*) *SNP*-haplotype and 7.5% were linked to the (*a59*) haplotype. Similarly, in the AA cohort, of all the DRB1*15:01~DQB1*06:*02 HLA* Class II haplotypes present, 96% were linked to the (*a1*) haplotype. In the WTCCC, 99% of the DRB1*15:01~DQB1*06:*02* haplotypes were linked to the (*a1*) *SNP*-haplotype. However, in rare instances in the WTCCC, it was linked to other *SNP*-haplotypes [29]. The same was true for both of these *HLA* Class II motifs in the AA cohort. Many of these rare alternative linkages in both populations were shared across these two *HLA* haplotypes including (*a1*), (*a34*), (*a36*), (*a43*), and (*a71*). In the predominantly European WTCCC, (*a59*) was not linked to (*H+*) but rather to DRB1*01:02~DQB1*05:*01* [29]. In addition, in the WTCCC controls [29], 99%% of the DRB1*03:01~DQB1*02:*01* Class II haplotypes are linked either to the (*a6*) *SNP*-haplotype (84%) or to the (*a2*) *SNP*-haplotype (15%). By contrast, in the AA controls only 83% of these haplotypes are linked to one or the other of these two *SNP*-haplotypes and they are distributed quite differently– 53% linked to (*a2*) and 30% linked to (*a6*)–the opposite of the distribution in Europeans. The remaining 17% of these DRB1*03:01~DQB1*02:*01* haplotypes are, thus, linked to other, non-(*a2*) and non-(*a6*), *SNP*-haplotypes. Again, of these most frequent *CEHs* with these linkages, (*c3707*)–linked to (*a2*)–is African in origin whereas (*c1*) and (*c14*)–linked to (*a6*)–are European (*Fig 1*).

The *ORs* for the most frequent *CEHs* in our AA cohort are presented in *Tables 1 & 2*. Only 11 of these *CEHs–*(*b1*), (*c1*), (*c2*), (*b2*), (*b3*), (*b4*), (*c6456*), (*c3*), (*b7*), (*c3707*), and (*b6*)–had more than 20 representations available in the AA cohort and, only 3 of these *CEHs*–(*b1*), (*c1*), and (*c2*)–had more than 40 representations available. Also, among these 11 most frequent *CEHs*, (*b3*), (*b7*), and (*b6*) carried the DRB1*15:03~DQB1*06:*02~a36* haplotype and (*c2*) and (*c3*) carried the (*H+*) haplotype. These are the haplotypes considered in our primary analysis (*Fig 1*). The only unequivocally significant association in the AA cohort (compared to neutral reference–see Methods), among individuals who didn’t carry any (*H+*) haplotypes, was for the possession of a single copy of the (*c2*) *CEH* (*OR = 3*.*30; p<0*.*0001*). There was only one individual in the AA cohort who possessed two copies of the (*c2*) *CEH* so the association for the homozygous state could not be tested. Moreover, the magnitude of this single copy association is the same as that found for possession of a single copy of (*c2*) in the predominantly European WTCCC (*OR = 3*.*25; p<10*^*−168*^)–compared to a neutral reference (see Methods). In addition, as shown in *Tables 1 & 2*, four other haplotypes–(*b1*), (*b3*), (*b4*), and (*c6666*)–had marginal associations (*p = 0*.*05–0*.*10*). In contrast to (*c2*), all of these *CEHs* were relatively “protective” compared to a neutral reference (*Tables 1 & 2*). The only *CEHs* carrying the DRB1*03:01~DQB1*02:*01* Class II motif that also had more than 20 representations were (*c1)* and (*c3707*) and these associations for single copy carriers were not significant (*Table 2*). Adjustments for admixture and population stratification did not alter any of these findings. However, if interaction terms are included in the regression equations, the associations for (*b1*), (*b3*), (*b4*), and (*c6666*) each become nominally significant (*p = 0*.*01–0*.*05*). Nevertheless, regardless of these statistical uncertainties, several of our observations conform to what has been demonstrated previously [29, 31]. For example, the *OR* for both (*c2*) and (*c6*) are greater than that for the (*c3*) *CEH* (*Table 1*); possession of a single copy of (*c5*) is relatively “protective” among non-(*H+*)-carrying individuals (*Table 2*); and the *OR* for (*c5*) was significantly less (*p = 0*.*003*) than that for (*c2*) and trended (*p = 0*.*06–0*.*13*) in the same direction for (*c6*) and (*c3*), respectively.

It is important also to consider how the various *CEHs* differ from each other with respect to their disease association rather than focusing solely on how each differs from any specific reference population. Thus, considering *CEHs* that carry the DRB1*15:03~DQB1*06:*01~a36* Class II motif, the (*b3*) *CEH* was significantly “protective” (*p = 0*.*02*) compared to the (*b6*) and (*b7*) *CEHs* (*Table 3*). Similarly, (*c2*) is associated with significantly more risk (*p<10*^*−5*^) than the (*b3*) *CEH*. In the case of (*c3*), the risk was significantly greater (*p = 0*.*01*) than (*b3*). Also, combining those *CEHs*, which that share their *HLA* Class II haplotypes, the *OR* for the (*H+*) haplotype in the AA cohort is greater than that for the DRB1*15:03~DQB1*06:*02~a36* haplotype (*p = 0*.*004*). Also, the combination of these two *HLA* Class II haplotypes into the same genotype did not seem to result in any increased “risk” of MS (*OR = 1*.*8; CI = 0*.*9–4*.*0*) compared to either haplotype alone. And finally, the *OR* for the (*H+*) haplotype in the WTCCC is significantly greater than that for the (*H+*) haplotype in the AA cohort (*p = 0*.*01*).

**Table 3:** Comparisons between different *CEHs* carrying the Class II motif of either DRB1*15:01~DQB1*06:*02~a1* or DRB1*15:03~DQB1*06:*02~a36* *.

				*1503–0602~a1*		*1503–0602~a36*	
				c2	c3	b3	b6
			***OR1***	**3.30**	**1.49**	**0.50**	**1.88**
		***OR1***	***SE***	**0.32**	**0.41**	**0.36**	**0.46**
***1503–0602~a1***	**c3**	**1.49**	**0.41**	2.2 (0.8–6.1)			
***1503–0602~a36***	**b3**	**0.50**	**0.36**	6.5 (2.6–16.8) ****	3.0 (1.0–8.6) ^*^		
	**b6**	**1.88**	**0.46**	1.8 (0.6–5.3)	0.8 (0.2–2.7)	0.3 (0.1–0.9) *	
	**b7**	**2.01**	**0.46**	1.6 (0.5–4.9)	0.7 (0.2–2.5)	0.3 (0.1–0.8) *	0.9 (0.3–3.4)

* Comparisons of the odds ratio (*OR1*) for the different *CEHs* listed in the *Table*. The numbers in in parentheses represent the 95% confidence intervals, at the point of intersection, the *OR1* in the column to that in the row. Only *CEHs* having more than 20 representations in the AA cohort are compared.

**Fig 3:**
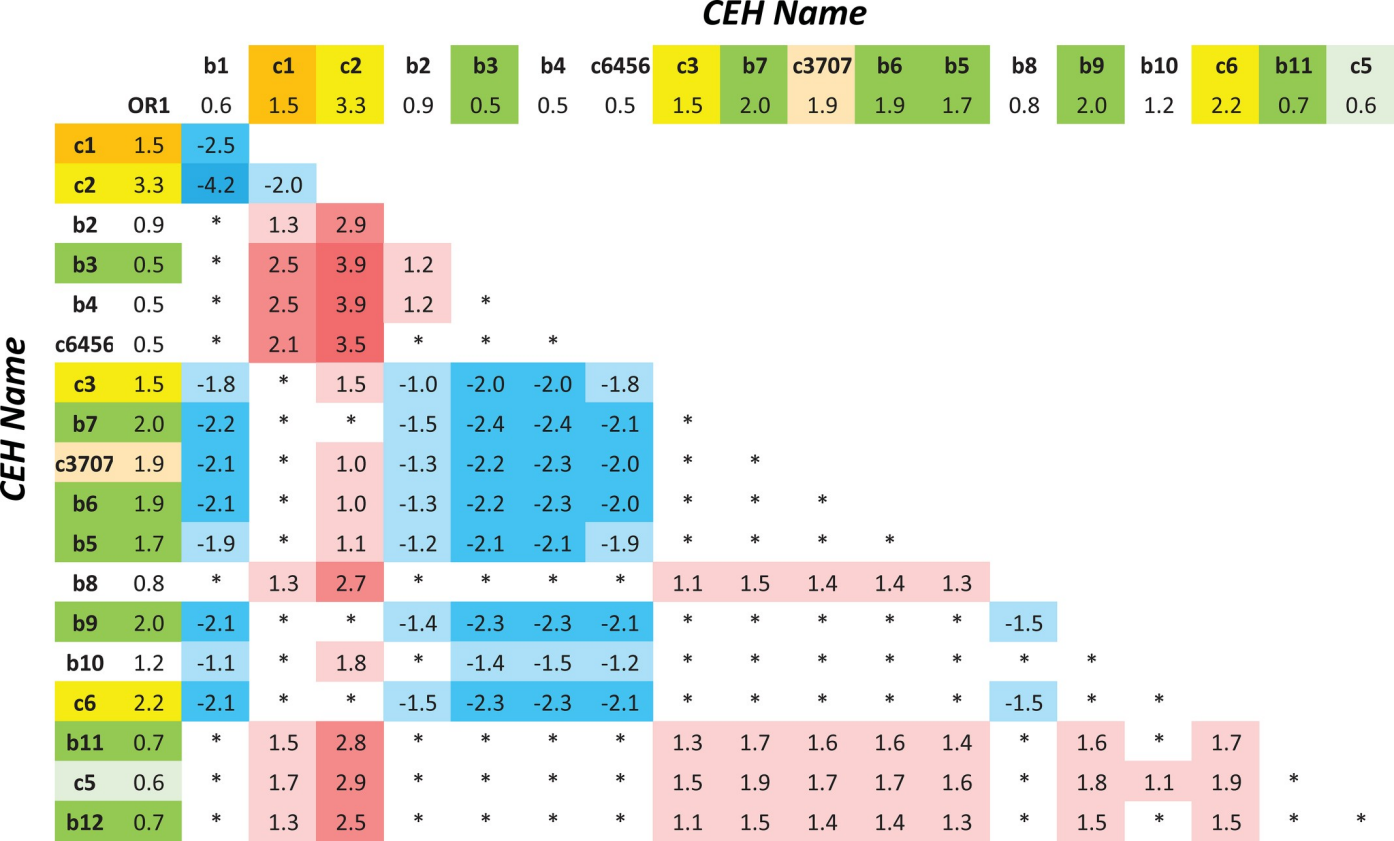
Odds ratio (*OR1*) of disease were calculated for individuals having only 1 copy of the listed *CEH* among individuals who have no (or no other) copies of an (*H+*) carrying *CEH*. Comparisons of the *OR1*s for the different *CEHs* listed in the *Table*. The numbers in the *Table* represent the *z-scores* comparing, at the point of intersection, the *OR1* in the column to that in the row. Positive numbers indicate that the *OR1* for the column *CEH* is greater than that for the row. Absolute *z-values* (|*z|* ≥ 3.0) are highlighted in dark blue (negative) or dark red (positive); absolute *z-values* (2.0 ≤ |*z|* < 3.0) are highlighted by medium blue (negative) or medium red (positive); absolute *z-values* (1.0 ≤ |*z|* < 2.0) are highlighted in light blue (negative) or light red (positive); absolute *z-values* (|*z|* < 1.0) are indicated by asterisks. *CEHs* carrying the (*H+*) haplotype are highlighted in yellow; *CEHs* carrying the DRB1*15:01~DQB1*06:*02~a36* haplotype are highlighted in green; *CEHs* carrying the DRB1*03:01~DQB1*02:*01~a6* haplotype are highlighted in orange; *CEHs* carrying the DRB1*03:01~DQB1*02:*01~a2* haplotype are highlighted in light orange; *CEHs* carrying the DRB1*04:01~DQB1*03:*01~a3* haplotype are highlighted in light green. Benjamini-Hochberg adjustment requires (|*z| >* 3.3) for significance. Because the *CEHs* are listed in descending order of their frequency, comparisons in the upper left-hand side of the *Figure* have the greatest statistical power.

The size of our AA cohort was quite small so that most of the *CEHs* had a very low number of representations in the dataset. Thus, despite their high (percentage-wise) frequencies (*see Tables 1 and 2*), the statistical power for most individual *CEH* comparisons was quite limited. At best, therefore, the potential *CEH*-comparisons in our AA cohort, other than the comparisons of primary interest, can provide only exploratory point-estimates for any possible relationship (*see* Methods). These comparisons are shown in *Fig 3* for all *CEHs* in our AA cohort that have more than 15 representations available. Despite the lack of statistical power, however, it seems clear from *Fig 3* that, in general, *ORs* for the (*b1*) (*b3*), (*b4*), and (*c6456*) *CEHs* are notably smaller than the *ORs* for the (*c1*), (*c2*) (*c3*), (*b7*), (*c3707*), (*b6*) (*b5*), (*b9*), and (*c6*) *CEHs*. We previously undertook a more fine-grained analysis of such relationships as these in the predominantly European WTCCC data [29]. However, in that study, we were able to consider only *CEHs* that had at least 50 representations (and many with hundreds) in the dataset–a circumstance that gave us a statistical power, which was not possible in a cohort of this size.

## Discussion

The present study provides considerable insight both to the haplotypic composition of an African American population and to the relationship that this composition has to MS-susceptibility. Indeed, of the 32 most frequent (independently phased) *CEHs* in our AA cohort, 27 (84%) of them were identical to *CEHs* (also independently phased) in African and/or European populations [28, 29]. Moreover, of these 18 were clearly of African origin, 9 were clearly of European origin. The remaining 5 *CEHs*–(*b5*), (*b10*) (*b14*), (*b16*), and (*b18*)–were probably also African in origin (*see* Results)–a circumstance that fits well with the average admixture (73%) observed in our cohort. The high frequency of these particular *CEHs* in our AA cohort (*Tables 1 and 2*), therefore, seems likely to be due to their high frequency in certain sub-populations of Africa, which were not well-represented in the African controls of Gragert and colleagues [28]. As a result, all 32 of these most-frequent *CEHs* seem to have remained remarkably intact over the period of time (<600 years), during which the admixture of our AA cohort was taking place. This observation underscores the stability of this *CEH* composition over relatively short time-intervals. By contrast, the considerable variability of *CEH* composition between African, European, and other populations [27–31] indicates a the *CEH* composition of different populations must be remarkably divergent over much longer periods of time. Presumably, such divergence is due to specific environmental and/or biological pressures that vary with time, with geographic location, or with both [26–31].

Among individuals who either don’t carry any (*H+*) haplotypes, or don’t carry any other (*H+*) haplotypes, we noted that some *CEHs* seem to be “protective” (e.g., *b1*, *b3*, *c5*), whereas others seem to carry “risk” (e.g., *c2*, *c3*, *b6*, *b7*). However, this distinction is simply a matter of definition. As a purely hypothetical example, we can arbitrarily designate one of two different haplotypes in some genomic region as “*A*” haplotypes and the other as “*B*” haplotypes. In this circumstance, any “protective” effect in individuals carrying “*A*” haplotypes compared to a reference group of individuals carrying “*B*” haplotypes is equivalent to a “risk” effect in individuals carrying “*B*” haplotypes compared to a reference group of individuals carrying “*A*” haplotypes. Thus, any notion of “risk” or “protective” haplotypes depends completely upon risk ratio between each haplotype being considered and the reference group chosen [31, 36–39]. By contrast, when two *ORs* are directly compared to each other as an estimate of the relative risk ratio, any chosen reference group becomes irrelevant [31, 36–39]. This point is critical when assessing in MS-susceptibility because, as noted earlier, more than 92.7% of individuals have no risk of MS whatsoever [5] and, using this group as the reference, even the group of individuals who don’t carry the (*H+*) haplotype will have an infinite relative risk. Consequently, it is the relative risk ratios (*Fig 1*) that provide the most reliable information regarding susceptibility.

Understanding this and even using this small dataset, it is clear that *CEH* composition has an important impact on MS-susceptibility in an African American population, much as it does in the predominantly European WTCCC [29]. Thus, the strongest statistical association with MS in both populations was for the (*c2*) *CEH*, which carries an (*H+*) Class II haplotype in addition to its Class I haplotype (*Table 1; Fig 1*). Moreover, the degree of risk associated with this predominantly European *CEH* (when compared to a similar reference group) was the same in each population (*OR ≈ 3*.*3*). Despite this, the (*H+*) haplotype, overall, appeared to be associated with less “risk” in African Americans (*p = 0*.*01*). However, this observation may be an artifact of combining, into a single group, different *CEHs*, many of which are known to carry different risks and which have different relative frequencies in the two populations [29]. For example, in the WTCCC cohort [29], the odds of disease for the *c3 CEH* was (*OR =* 2.2; p<10^−38^), which was significantly smaller (*p<*10^−6^) than that observed for the *c2 CEH* (*OR =* 3.25; *p<*10^−168^). Similarly, the AA cohort, the odds of disease for the *c3 CEH* (*OR =* 1.5; ns) was smaller than that for the *c2 CEH* (*OR =* 3.3; *p<*10^−4^).

Also, the apparent risk difference between DRB1*15:03~DQB1*06:*02~a36* and (*H+*) is likely explained, at least partly, in a similar manner. For example, the (*b3*) *CEH* is carries significantly less risk than (*b7*) and (*b6*) and, possibly, the (*b11*) and (*b12*) *CEHs* as well (*Table 3*; *Fig 3*). Therefore, combining all of these *CEHs* into a single group will lead to an intermediate assessment of risk (which it did). In fact, because the relationship between a specific *CEH* and MS depends upon the nature of the entire haplotype (*Table 3*), the relationship between the *HLA* Class II portion of a *CEH* and MS, will, necessarily, be heterogeneous [29].

Other investigators have also explored the differential MS susceptibility in Africans and Europeans. For example, in a cohort of African Americans, Chi and coworkers [40] reported that the MS-risk *OR* for HLA*DRB1*15:*01* allele of European origin was three times that for the same allele of African origin. In addition, these authors found that there were differences between these alleles in the amino acid composition, especially in the region of exon 1, but also in the regions of exons 3 and 5 [40]. Because exon 2 codes for the extracellular loop of the *DRB1* protein, which contains the antigen recognition site (*ARS*), there were no differences found in this exon between African and European versions of this protein [40]. The authors raised the possibility that these differences could have functional consequences for the *DRB1* molecule, despite Europeans and Africans sharing the same *ARS* [40]. For example, potentially, alterations in the non-*ARS* regions of the protein might impact the transcription, the translation, or the expression of *DRB1* gene even if these changes didn’t impact the binding and recognition of antigen by the mature protein. This is an intriguing possibility although it should be noted that, even among Europeans, there are differences in risk between different DRB1*15:*01* alleles. For example, in the WTCCC, individuals who carry the (*H+*) haplotype (*OR = 3*.*0*) have almost twice the MS-risk (*p<10*^*−6*^) compared to individuals who carry other DRB1*15:*01* containing haplotypes (*OR = 1*.*6*). Also, as discussed in the *Introduction*, the same allele resides on many different *CEHs* and often these *CEHs* have very different disease associations, even among persons of very similar ancestry [29]. And, finally, because so few (*H+*) carriers are even susceptible to (i.e., have any chance of) getting MS, it is unclear how any single variant of the DRB1*15:*01* allele could possibly be responsible for the relationship between DRB1*15:*01* and susceptibility to MS [5]. This is especially true for the circumstance in which 94% of European DRB1*15:*01* alleles are identical [40].

In summary, the haplotypic (*CEH*) structure of our AA cohort is quite similar to the structure of other world populations [28, 29]. The *CEH* composition of our AA cohort appears to be an admixture of common *CEHs* of either African or European origin, which seem not to have been modified during the period of admixture. Moreover, those *CEHs*, which are likely of European origin (*Fig 1*), and which are associated with MS-risk in the predominantly European WTCCC cohort [29]–i.e., (*c1*), *(c2*), (*c3*), (*c5*), and (*c6*)–generally seemed to have a similar impact in our AA cohort (*Tables 1 & 2*). Of the common African *CEHs*, which carried the DRB1*15:03~DQB1*06:*02~a36* haplotype, many seemed to have an MS-risk, which exceeded that in a reference group of non-(*H+*)-carrying individuals. However, even with this haplotype, the actual risk (i.e., whether it was “protective’ or carried “risk”) depended upon the specific *CEH* being considered (*Table 2*). By contrast, most other common *CEHs* of likely African origin (*Tables 2&3*) seemed to be “protective” relative to this same reference group–a circumstance that might help to rationalize, at least partly, the lower risk of MS in African compared to European Americans. Nevertheless, even though the risk of MS may be less in African Americans, the disease may be more severe and the disability greater compared to European Americans [Cree 2004].

## Methods

### Ethics statement

This research has been approved by the University of California, San Francisco’s Institutional Review Board (IRB) and has been conducted according to the principles expressed in the Declaration of Helsinki.

### Study participants

The study population consisted of 1,305 patients with MS and 1,155 controls, all of whom self-identified as being African American (AA). The diagnosis of MS in this cohort was made based upon internationally recognized criteria [41–43]. The UCSF Institutional Review Board approved the protocol and written informed consent was obtained from each study participant.

For comparison purposes, we used the data from the WTCCC. The patients enrolled in this multinational cohort study were predominantly of European ancestry [13]. This cohort consists of 18,492 controls and 11,144 cases with MS and has been described in detail previously [13, 29]. The WTCCC granted data access for this study.

Also, for comparison, we analyzed the 28,557 native Africans and 1.24 million Europeans from the multinational data-set of Gragert et al. [28]. This study calculated six-locus high resolution *HLA*-*A~C~B~DRB3/4/5~DRB1~DQB1* haplotype frequencies using the “*Be the Match*” registry donors who volunteered to be typed by DNA methods at recruitment. Mixed resolution *HLA* typing data was inputted using a modified expectation–maximization (EM) algorithm in the form of genotype lists generated by interpretation of primary genomic typing data to the IMGT/HLA v3.4.0 allele list [28]. The full cohort consisted of 6.59 million subjects categorized at a broad level by race. In sum, 25.8% of the individuals were typed at the *C* locus, 5.2% typed at the *DQB1* locus, and all individuals were typed for the *A*, *B*, *& DRB1* loci. The purpose of this study was to improve match predictions regarding donor selection for hematopoietic stem cell transplantation.

### Genotyping, and quality control

The genotyping methods and quality control for the AA cohort has been described in detail previously [44]. Briefly, DNA was extracted from whole blood and *SNP* genotyping was conducted using the MS Chip, which is a custom genotyping array of Illumina Infinium. This array includes content designed to contain ancestry informative markers and other genetic markers specific interest for multiple sclerosis. Genotyping was done by the Center for Genome Technology (part of the John P. Hussman Institute for Human Genomics; University of Miami) and genotype calling was made using GenomeStudio v2.0. The identities of the five *HLA* alleles in the *MHC* region (*A*, *C*, *B*, *DRB1* and *DQB1*) were determined for each participant by imputation using the HIBAG method [45]. We built a custom reference panel using CAAPA data (dbGaP Study Accession: phs001123.v1.p1) to impute HLA alleles from African American ancestry as accurately as possible. We used best guess HLA alleles. The posterior probabilities cutoff was 0.5, as recommended by the original HIBAG authors [45]. The percentage of alleles with posterior probabilities (> 0.5) was: HLA-A: 98%; HLA-B: 82%; HLA-C: 95%; HLA-DRB1: 85%; HLA-DQB1: 98%.

The genotyping and quality control methods both for the WTCCC and for the study of Gragert et al. [28] have also been described in detail previously [13, 14, 16, 18, 19, 28].

### Estimating admixture

The ancestry of individuals in our AA cohort was inferred using ADMIXTURE software [46]. On chromosome 6, we selected *SNPs* (*n = 2504*), which overlapped between the AA individuals and two subsets of 1000 Genomes project (CEU, *n = 99*; YRI, *n = 108*), and which were representative of the European and African populations [47, 48].

### Data access

Due to limitations in the original signed consents and to IRB restrictions regarding patient confidentiality, we are unable to provide individual genotype data for our African American cohort. For further inquiries or information, individuals may contact the IRB Chair at UCSF (Victor I. Reus, MD) at https://irb.ucsf.edu/. Nevertheless, summary statistics for the *MHC SNPs* are available upon request from the authors of the original paper [32]. For access to the WTCCC data and the “*Be the Match*” registry data (which are not ours), the original authors should be contacted directly [13, 28]. Our group obtained the data as outlined above although, because the lead author and principal investigator of the original AA publication [32] was also our co-author (JRO), we had access to the individual AA genotype data.

### Statistical methods

#### Phasing

Both the phasing of alleles at each of five *HLA* loci (*HLA-A*, *HLA-C*, *HLA-B*, *HLA-DRB1* and *HLA-DQB1*) and the phasing of the *SNP*-haplotypes surrounding the Class II region of the *DRB1* gene were accomplished using previously-published probabilistic phasing algorithms [29, 49–51].

#### Haplotype frequencies and association testing

Disease association tests, as measured by *ORs* and confidence intervals (*CIs*) comparing cases to controls, were calculated for each of the *CEHs*. These *ORs* were determined relative to a so-called “*neutral reference group*”. For *CEHs* that did carry the (*H+*) motif, this reference group excluded all (*H+*) carriers. For *CEHs* that carried the (*H+*) motif, this reference group excluded all individuals who carries another copy of (*H+*). The AA data was considered in its entirety and not further stratified. The significance of the differences in *ORs* for disease association (comparing cases to controls) for any two haplotypes or genotypes was determined by *z-scores* calculated from the differences in the natural logarithm of the *ORs* such that:

$$z=[\ln(OR_{1})-\ln(OR_{2})]/\sqrt{{\left\{SE[\ln(OR_{1})]\right\}}^{2}+{\left\{SE[\ln(OR_{2})]\right\}}^{2}}$$

**Fig 4:**
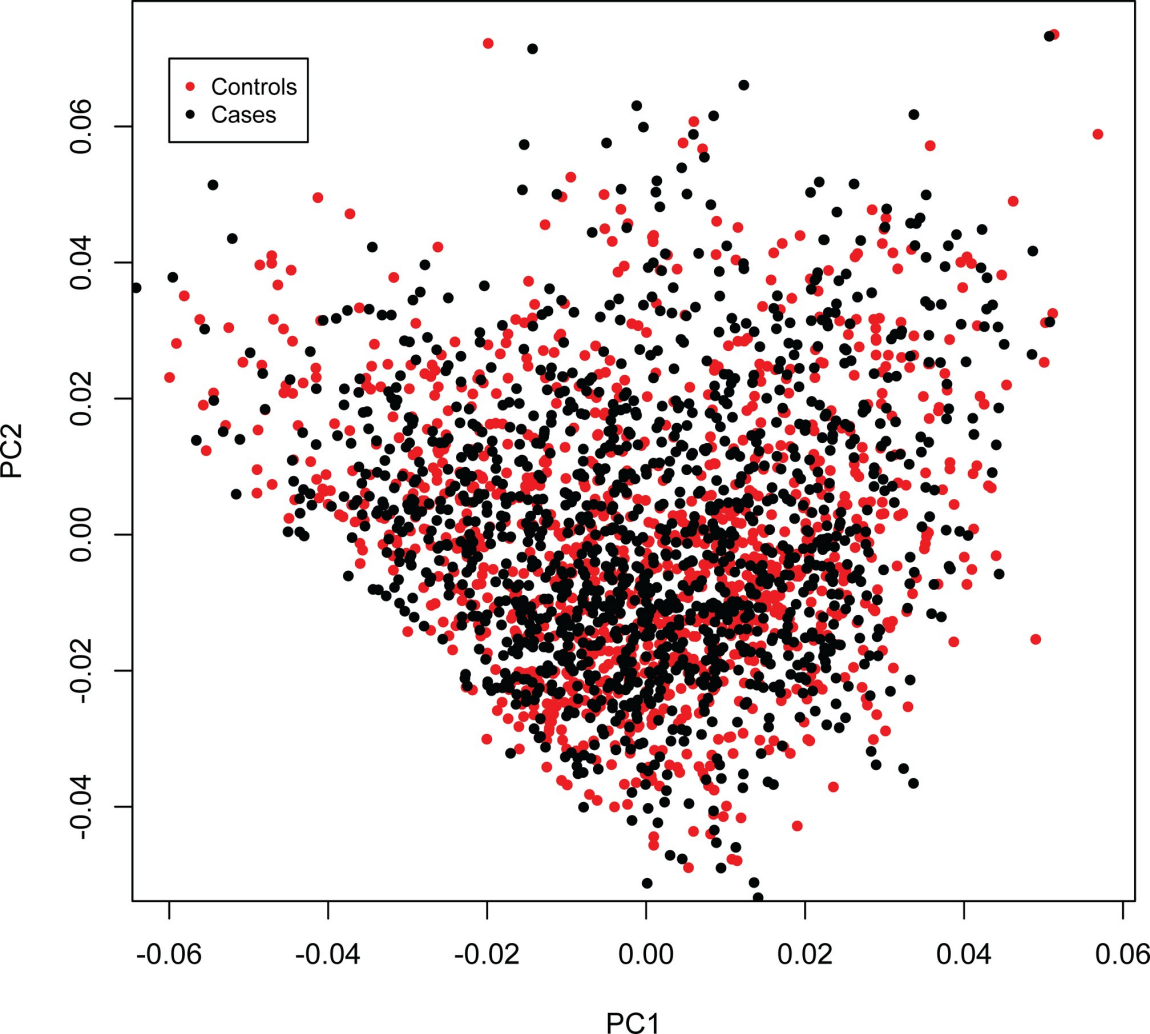
A principal components analysis of the pairwise “identity by decent” distances between cases and controls demonstrated no difference between cases and controls.

Benjamini-Hochberg method was used to correct for multiple testing of possible MS-association for the different *CEHs*. To maximize the statistical power to detect differences between *CEHs*, our primary analysis was on only those African American *CEHs*, which carried either the (*H+*) haplotype or the related DRB1*15:03~DQB1*06:*02~a36* haplotype, and which had more than 20 representations in our AA cohort. Other comparisons were included only to provide exploratory point-estimates. All *ORs* used for pair-wise comparisons within the *MHC* were estimated relative to a reference group that excluded individuals who either carried any (*H+*) haplotypes or carried any other (*H+*) haplotypes. Within the AA cohort, to assess population stratification, we performed a principal components (*PC*) analysis, which excluded *MHC SNPs* (Eigensoft) and used regression analysis to correct the observations in *Tables 1 & 2* for the possible effects of either population stratification or admixture within the AA cohort. In this analysis we used the first 10 of these *PC* components which accounted for 71% of the variance. Neither of these adjustments significantly altered any of our observations. Also, a PC analysis of the pairwise “identity by decent” distances demonstrated no differences between cases and controls (*Fig 4*).
